# Effect of BIM expression on the prognostic value of PD-L1 in advanced non-small cell lung cancer patients treated with EGFR-TKIs

**DOI:** 10.1038/s41598-023-30565-4

**Published:** 2023-03-09

**Authors:** Chang-Yao Chu, Chien-Yu Lin, Chien-Chung Lin, Chien-Feng Li, Shang-Yin Wu, Jeng-Shiuan Tsai, Szu-Chun Yang, Chian-Wei Chen, Chia-Yin Lin, Chao-Chun Chang, Yi-Ting Yen, Yau-Lin Tseng, Po-Lan Su, Wu-Chou Su

**Affiliations:** 1 Department of Pathology, Chi-Mei Medical Center, Tainan, Taiwan; 2 Department of Internal Medicine, National Cheng Kung University Hospital, College of Medicine, National Cheng Kung University, No.138, Shengli Road, North District, Tainan, 704 Taiwan; 3 Institute of Clinical Medicine, National Cheng Kung University Hospital, College of Medicine, National Cheng Kung University, Tainan, Taiwan; 4 Department of Biochemistry and Molecular Biology, College of Medicine, National Cheng Kung University, Tainan, Taiwan; 5 Department of Medical Research, Chi Mei Medical Center, Tainan, Taiwan; 6 Trans-Omic Laboratory for Precision Medicine, Precision Medicine Center, Chi Mei Medical Center, Tainan, Taiwan; 7 National Institute of Cancer Research, National Health Research Institutes, Tainan, Taiwan; 8 Department of Oncology, National Cheng Kung University Hospital, College of Medicine, National Cheng Kung University, Tainan, Taiwan; 9 Department of Medical Imaging, National Cheng Kung University Hospital, College of Medicine, National Cheng Kung University, Tainan, Taiwan; 10 Department of Surgery, National Cheng Kung University Hospital, College of Medicine, National Cheng Kung University, Tainan, Taiwan; 11 Center of Applied Nanomedicine, National Cheng Kung University, Tainan, Taiwan

**Keywords:** Oncology, Cancer therapy, Lung cancer

## Abstract

The role of Programmed Cell Death Ligand 1 (PD-L1) expression in predicting epidermal growth factor receptor tyrosine kinase inhibitor (EGFR-TKIs) efficacy remains controversial. Recent studies have highlighted that tumor-intrinsic PD-L1 signaling can be modulated by STAT3, AKT, MET oncogenic pathway, epithelial–mesenchymal transition, or BIM expression. This study aimed to investigate whether these underlying mechanisms affect the prognostic role of PD-L1. We retrospectively enrolled patients with EGFR mutant advanced stage NSCLC who received first-line EGFR-TKI between January 2017 and June 2019, the treatment efficacy of EGFR-TKI was assessed. Kaplan–Meier analysis of progression-free survival (PFS) revealed that patients with high BIM expression had shorter PFS, regardless of PD-L1 expression. This result was also supported by the COX proportional hazard regression analysis. In vitro, we further proved that the knockdown of BIM, instead of PDL1, induced more cell apoptosis following gefitinib treatment. Our data suggest that among the pathways affecting tumor-intrinsic PD-L1 signaling, BIM is potentially the underlying mechanism that affects the role of PD-L1 expression in predicting response to EGFR TKI and mediates cell apoptosis under treatment with gefitinib in EGFR-mutant NSCLC. Further prospective studies are required to validate these results.

## Introduction

Epidermal growth factor receptor (EGFR) mutation is the most common oncogenic driver gene in advanced non-small cell lung cancer (NSCLC), accounting for approximately 50% of patients^1^. The use of EGFR-tyrosine kinase inhibitors (TKIs) provides better progression-free survival (PFS) and objective response rate (ORR), it has become the mainstay treatment strategy in these patient populations^2^. However, approximately 20–30% of patients receiving EGFR-TKIs remain unresponsive^3^. The mechanism of primary resistance includes the activation of bypass or downstream signaling pathways^4^, presence of resistant kinase domain mutations (T790M mutation)^4^, histological transformation^5^, and deletion polymorphism of the Bcl-2 family member (BIM)^6^. Recently, there have been increasing studies regarding the role of the tumor microenvironment in the treatment efficacy of EGFR-TKIs, including the programmed death 1 (PD-1) and programmed death-ligand 1 (PD-L1) pathways^7^.

The role of PD-L1 in predicting treatment outcomes to EGFR-TKIs remains controversial; some cohort studies have demonstrated that high PD-L1 expression is associated with poor treatment efficacy of EGFR-TKIs^8–15^. Other studies have shown that PD-L1 expression levels do not affect the efficacy of EGFR-TKIs^16–19^. Current studies had highlighted the multiple aspects of tumor PD-L1 signaling, including cell-intrinsic effects and cell-extrinsic PD-L1 signaling^20^. The cell-extrinsic PD-L1, high expressed PD-L1 on the surface of cancer or immune cells, is pathogenic primarily by inhibiting CD8+ cytotoxic T cells and contributes to the development of immune checkpoint blockade in treating NSCLC. In contrast, cell-intrinsic PD-L1 signals originate from PD-L1 in the surface, cytosol, and nucleus by post-translational modifications and can be elicited by a PD-1-independent pathway. It can be upregulated by activated EGFR through the interleukin 6 (IL6)/Janus kinase (JAK)/signal transducer and activator of transcription 3 (STAT3) signaling pathway in NSCLC cells^20,21^. Moreover, the downstream pathway of EGFR and STAT3 not only directly binds to the promoter to upregulate the transcriptional expression of PD-L1^22^ but also mediates resistance to EGFR-TKI via the Zeb1 activity of the EMT pathway^23^. AKT activation also triggers PD-L1 expression in EGFR-mutant lung cancer cell lines^24^ and becomes a convergent feature of acquired resistance to EGFR-TKIs^25^. Additionally, epithelial-mesenchymal transition (EMT) could upregulate PD-L1 expression via an NF-κB-dependent mechanism^26^. Moreover, the expression of PD-L1 was also associated with increased BIM expression^27^, which is an important factor in EGFR-TKI-mediated apoptosis^28^.

Since these pathways both mediate PD-L1 expression and are associated with primary resistance to EGFR-TKI, we conducted this study to identify the key pathway that may impact the inconsistency in the prognostic value of PD-L1 in advanced non-small cell lung cancer patients treated with EGFR-TKIs.

## Methods

### Patient characteristics

Patients with EGFR-mutant advanced-stage NSCLC who received first-line EGFR-TKIs at a tertiary referral center were retrospectively reviewed from January 2017 to June 2019. At the initial diagnosis, all patients underwent imaging examinations for complete staging, including computed tomography of the chest, brain magnetic resonance imaging, and bone scan. Staging was performed according to the American Joint Committee on Cancer 8th edition. Baseline characteristics, including age, sex, stage, performance status, presence of brain metastasis, EGFR mutation subtype, and choice of first-line EGFR-TKI therapy, were recorded. After the initiation of EGFR-TKI treatment, all patients underwent computed tomography (CT) of the chest to evaluate treatment response every 3 months until disease progression. The radiological response was defined using the Response Evaluation Criteria in Solid Tumors (RECIST) version 1.1^29^. The study protocol was reviewed and approved by the Review Board and Ethics Committee of National Cheng Kung University Hospital (NCKUH B-ER-107-374). Given the retrospective nature of the current study, the need of informed consent was waived by the Review Board and Ethics Committee of National Cheng Kung University Hospital. All the study protocol of this study followed the Declaration of Helsinki guidelines.

### Immunohistochemical staining and scoring for PD-L1

Immunohistochemical (IHC) staining of PD-L1 expression on tumor cells was assessed using the PD-L1 Clone 22C3 pharmDx kit (Dako; Agilent Technologies, Inc., Santa Clara, CA, USA) and the Automated Link 48 platform (Dako, Carpinteria, CA). The percentage of positive membrane staining of PD-L1 was calculated after the evaluation of at least 100 viable cells, which was referred to as the tumor proportional score (TPS)^30^. TPS was categorized into three groups (no expression with TPS of < 1%, low expression with TPS of 1–49%, and high expression with TPS of 50–100%), which used 22C3 antibodies as a companion diagnostic test^31,32^. The stained tissue sections were independently scored by Dr. Chang-Yao Chu at Chi-Mei Medical Center and other pathologists at NCKUH who were blinded to the patients’ clinical characteristics and outcomes.

### Immunohistochemical staining of other biomarkers

Immunohistochemical staining for p-STAT3, p-AKT, MET, E-cadherin, and BIM was performed using the streptavidin-peroxidase method. Briefly, 4-μm-thick sections were deparaffinized, rehydrated, and treated with 0.3% H_2_O_2_ to block endogenous peroxidase activity. Following rehydration through graded concentrations of ethanol and autoclave, nonspecific binding sites were blocked with 10% normal goat serum. The sections were then incubated at 4 °C overnight with the following antibodies: rabbit antibodies against human p-STAT3 (Cell Signaling Technology, #4060, dilution 1:100), p-AKT (Cell Signaling Technology, catalog #9145, dilution 1:100), MET (Abcam, catalog #Ab227637, dilution 1:30), E-cadherin (Cell Signaling Technology, catalog #3195, dilution 1:50), and BIM (Cell Signaling Technology, catalog #2933, dilution 1:100). The sections were then incubated with biotinylated peroxidase-labeled anti-rabbit antibody (DakoCytomation, catalog #K4003) for 30 min, followed by incubation with streptavidin–biotin peroxidase complex solution. The chromogen 3,3′-diaminobenzidine tetrahydrochloride was used. The expression of p-STAT3, p-AKT, MET, and BIM was defined in samples as any intensity of antibody staining and ≥ 1% of the tumor^33–36^. Samples with no E-cadherin membranous staining in any percentage of the tumor were categorized as a loss of expression, while those with E-cadherin membranous staining were categorized as having preserved expression^37^. Finally, the sections were lightly counterstained with hematoxylin. The stained tissue sections were independently scored by Dr. Chang-Yao Chu at Chi-Mei Medical Center and other pathologists at NCKUH who were blinded to the patients’ clinical characteristics and outcomes.

### Statistical analysis

The frequencies and descriptive statistics of the demographic and clinical variables were calculated. Categorical variables were compared using the chi-square test or Fisher’s exact test, whereas continuous variables were compared using the Student’s t-test or Wilcoxon rank-sum test. PFS was determined as the time period from initiation of EGFR-TKI therapy to radiological progression or death, which was estimated using the Kaplan–Meier method and compared with the log-rank test. The p-value was used to indicate statistical significance (P < 0.05). The Cox proportional hazards regression analysis was also performed to determine the determinants of PFS. The Cox proportional hazards were estimated using the formula:

$$h\left(t\right)={h}_{0}{(t)}^{{b}_{1}{X}_{1}+{b}_{2}{X}_{2}+\dots +{b}_{p}{X}_{p}}$$

where *t* represents survival time and $h\left(t\right)$ is the expected hazard at time *t*. The hazard function was determined by a set of determinants (X_1_, X_2_,…,X_p_). The coefficients (b_1_, b_2_,…,b_p_) represent the impact of each determinants. The ${h}_{0}\left(t\right)$ is the baseline hazard and represents the hazard when all the determinants are equal to zero. The possible determinants included prognostic factors based on prior studies^38,39^ and the IHC staining results of the current study. Statistical Analysis System^®^ software (version 9.4; SAS Institute, Cary, NC, USA) was used to perform analyses.

### Cell culture and transfection of siRNA

The HCC827 cell line was grown in RPMI-1640 medium (Thermo Fisher Scientific, Waltham, MA, USA) with supplement of 10% (v/v) fetal bovine serum (FBS) (Gibco; Life Technologies, Carlsbad, CA, USA) at 37 °C in an incubator with 5% carbon dioxide. The cells were maintained by monolayer culture and passaged twice or thrice per week. For siRNA transfection, HCC827 cells were cultured in a 6-cm dish. Thereafter, the cultured cells were washed once with sterile phosphate-buffered saline and the culture medium was supplemented with Lipofectamine (Dharmacon, Horizon Discovery, Lafayette, CO, USA) plus 100 nM of the ON-TARGETplus siRNA targeting CD274 or BCL2L11 (Dharmacon, Horizon Discovery, Lafayette, CO, USA). For drug treatment, gefitinib (Selleck Chemicals LLC, Houston, TX, USA) was dissolved in DMSO and diluted to the desired final concentrations with a growth medium immediately before administration. After 24-h transfection, the culture medium was replaced with a medium containing 1 μM or 5 μM gefitinib for the following 24 h.

### Assessing apoptotic ratio via flow cytometry

The flow cytometry was performed to assess the apoptotic fractions of cancer cell line. After 24-h drug treatment, the cells were trypsinized and stained with 5 μL FITC-Annexin V stock solution (#556547; BD Biosciences Pharmingen, CA, USA) and 5 μL propidium iodide (PI) stock solution (#550825; BD Biosciences Pharmingen, CA, USA). Then, the samples were incubated in the dark for 15 min, followed by the addition of a 400 μL Annexin V Binding Buffer (#422201, BioLegend, CA, USA) for the subsequent measurement. Both fluorochromes were excited by an argon-ion laser at 488 nm. FITC-Annexin V was measured on FL1 channel (filter: 530/30 nm) and PI was measured on the FL2 channel (filter: 585/42 nm). Total 1 × 10^4^ events were evaluated in each sample via flow cytometry (CytoFLEX Flow Cytometry, Beckman Coulter, Brea, CA, USA). Further analysis of the apoptotic ratios was implemented by CytExpert 2.2 (Beckman Coulter, CA, USA).

### Genomic testing of the BIM intronic deletion polymorphism

In patients with sufficient residual tissue, the genomic DNA was extracted from formalin-fixed paraffin-embedded (FFPE) biopsy slides using iCatcher FFPE Tissue DNA Kit (CatchGene Co., Ltd.). BIM polymorphic genotyping was performed as previously described^40^. Briefly, two separate polymerase chain reactions (PCRs) were performed for each sample, to determine the presence of the wild-type and deletion alleles using the forward primer 5′-CCACCAATGGAAAAGGTTCA-3′ for both wild-type and deletion alleles, the reverse primers 5′-CTGTCATTTCTCCCCACCAC-3′ and 5′-GGCACAGCCTCTATGGAGAA-3′ for the wild-type and deletion alleles, respectively. The PCRs were performed with the following thermo cycling conditions: 95 °C for 5 min; 39 cycles of 95 °C for 50 s, 58 °C for 50 s, and 72 °C for 1 min; and 72 °C for 10 min. Finally, the PCR products were analyzed on a 2% agarose gel, with 284 bp for the deletion alleles and 362 bp for the wild-type alleles.

## Results

### Patient demographics

**Figure 1:**
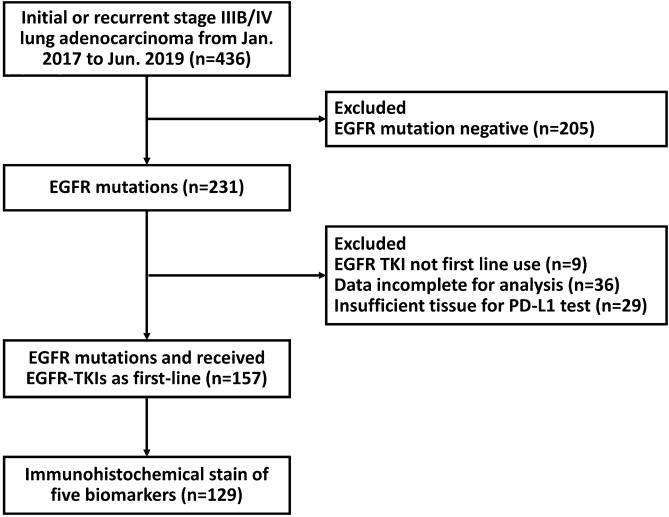
Patient flowchart.

Of the 231 patients with advanced EGFR-mutated NSCLC recruited between January 2017 and June 2019, 157 had sufficient tissue for PD-L1 evaluation. Among these patients, 129 underwent immunohistochemical staining for an additional five biomarkers. Figure 1 shows the flowchart for enrolling the subjects. The Table 1 had summarized the baseline characteristics of 157 patients. All the patients had histological adenocarcinomas and advanced-stage disease. The median age of the patients was 66 years (range, 61–75 years), and there were 61 men (38.9%) and 96 women (61.1%). Sixty-eight patients (43.3%) had brain metastases. The EGFR genotyping test revealed exon 19 deletion mutations in 74 (47.1%), L858R mutations in 74 (47.1%), uncommon mutations in 6 (3.8%), and complex mutations in 3 (2.0%) patients with NSCLC. Immunohistochemical staining of PD-L1 revealed no expression (< 1%) in 95 patients (60.5%), low expression (1–49%) in 42 patients (26.8%), and high expression (≥ 50%) in 20 patients (12.7%).

**Table 1:** Patient characteristics.

Characteristic	Total patients (%) N = 157
Age	
Median (range), years	66 (61–75)
< 65 years	67 (42.7%)
> 65 years	90 (57.5%)
Sex	
Female	96 (61.1%)
Male	61 (38.9%)
Brain metastasis	68 (43.3%)
Stage	
3B	8 (5.1%)
4A	40 (25.5%)
4B	109 (69.4)
Tumor size	
< 3 cm	32 (20.4%)
≥ 3 cm	125 (79.6%)
Nodal stage	
N0-2	48 (30.6%)
N3	109 (69.4%)
Performance status	
ECOG 0–1	138 (87.9%)
ECOG ≥ 2	19 (12.1%)
EGFR mutation	
Exon 19 deletion	74 (47.1%)
Exon 21 L858R substitution	74 (47.1%)
Uncommon mutation	6 (3.8%)
Complex mutation	3 (2.0%)
EGFR-TKI	
Gefitinib	22 (14.0%)
Erlotinib	67 (42.7%)
Afatinib	68 (43.3%)

EGFR, epidermal growth factor receptor; TKI, tyrosine kinase inhibitor.

### The PFS based on different PD-L1 and biomarker expression

**Figure 2:**
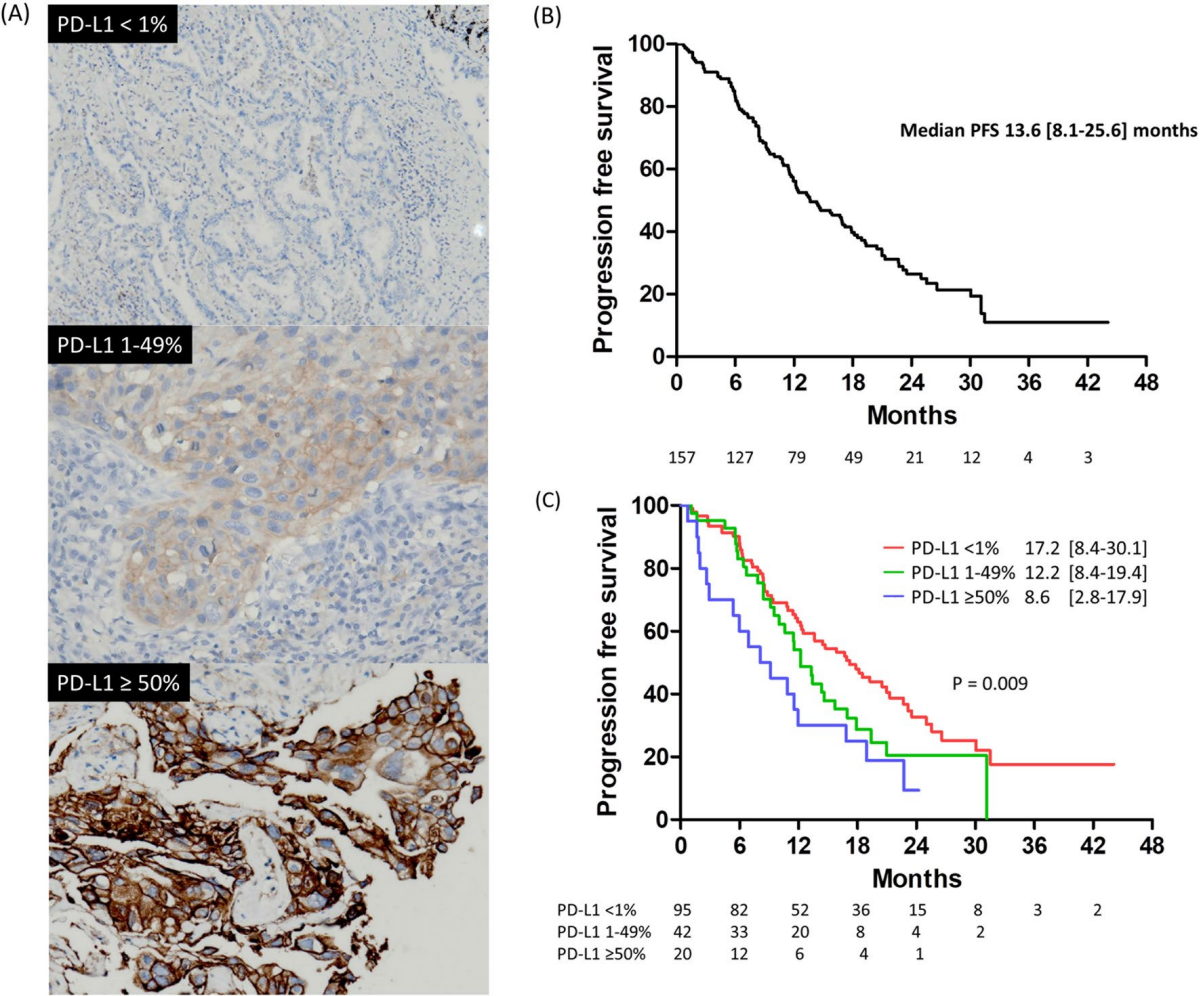
(**A**) The immunohistochemical stain of different PD-L1 expression levels. (**B**) Progression-free survival among EGFR-mutant NSCLC patients receiving first-line EGFR-TKI. (**C**) Kaplan–Meier analysis of progression-free survival among patients with different PD-L1 expression levels.

**Figure 3:**
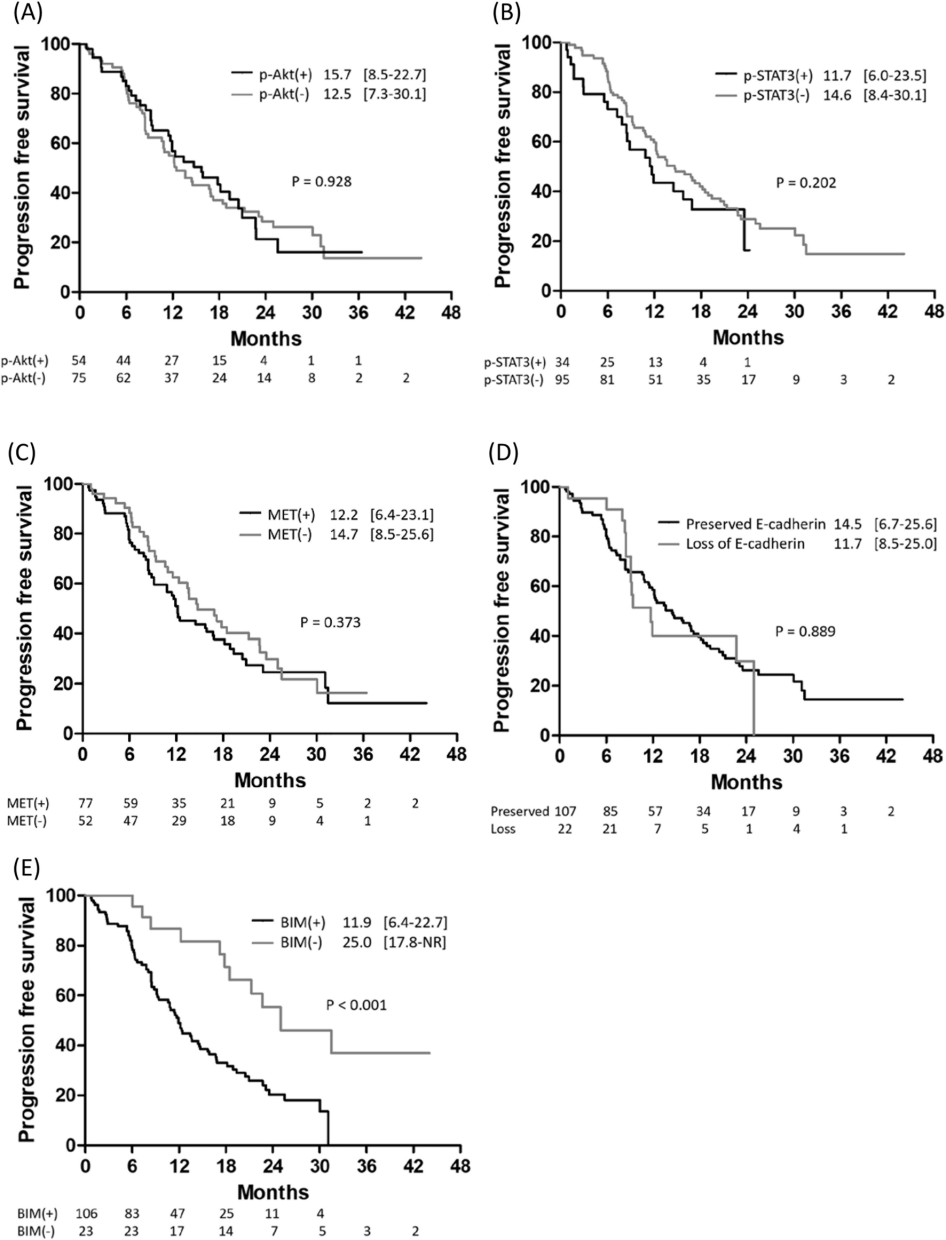
Kaplan–Meier analysis of progression-free survival among patients with the different expression levels of p-AKT (**A**), p-STAT3 (**B**), MET (**C**), E-cadherin (**D**), and BIM (**E**).

**Figure 4:**
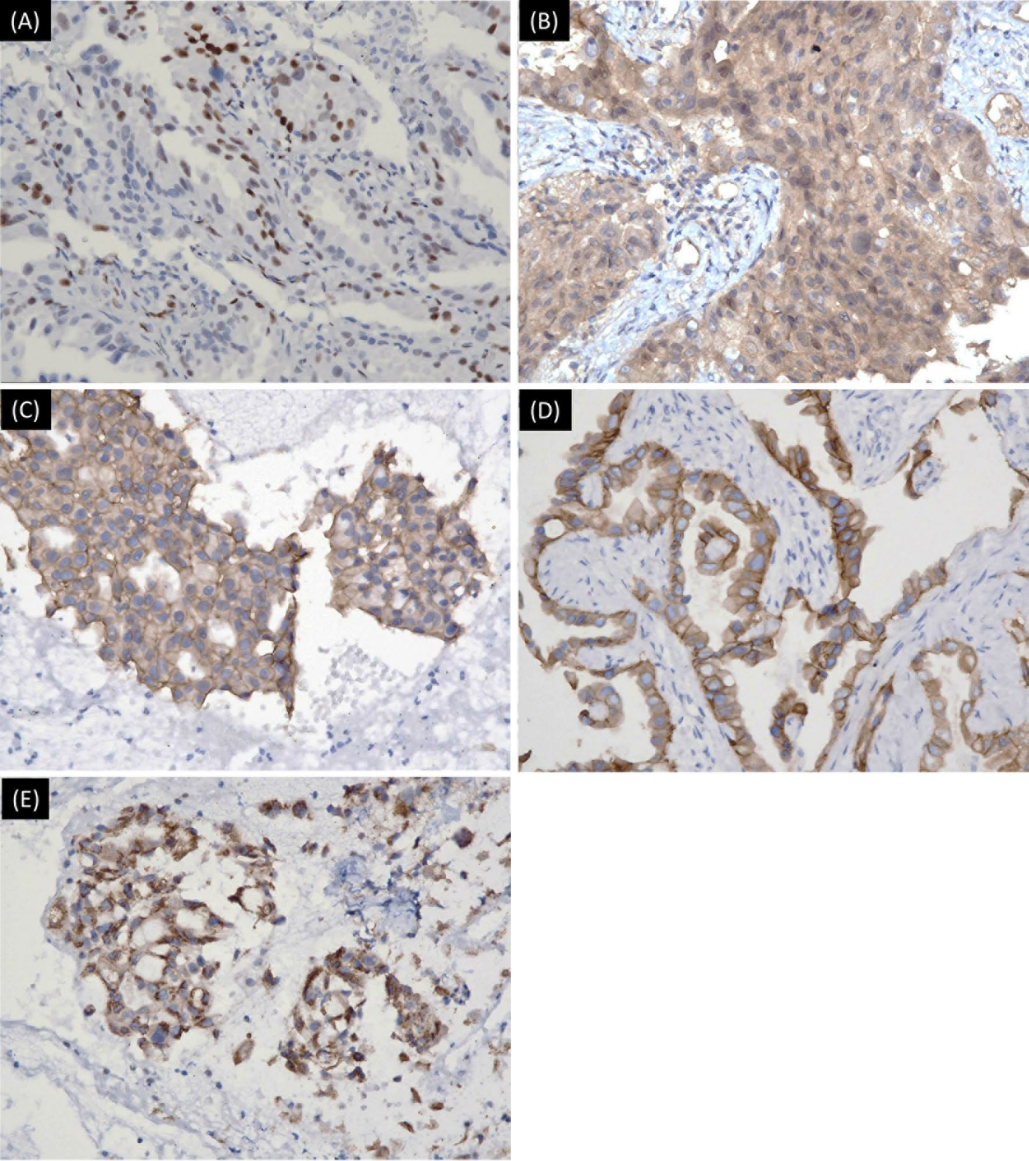
The immunohistochemical stain of p-AKT (1:100) (**A**), p-STAT3 (1:100) (**B**), MET (1:30) (**C**), E-cadherin (1:50) (**D**), and BIM (1:100) (**E**).

The immunohistochemical staining of different PD-L1 expression levels is presented in Fig. 2A. The median follow-up period was 19.1 months (interquartile range: 11.6–25.8). The median PFS among the 157 patients was 13.6 months (interquartile range: 8.1–25.6) (Fig. 2B). When patients were classified based on PD-L1 expression level, the patients with no PD-L1 expression had PFS of 17.2 months (interquartile range: 8.4–30.1), which was significantly longer than that of patients with low PD-L1 expression (median PFS 12.2 months, interquartile range: 8.4–19.4) and patients with high PD-L1 expression (median PFS 8.6 months, interquartile range, 2.8–17.9) (log-rank p = 0.009, Fig. 2C). Moreover, we evaluated the differences in PFS between patients with different biomarker expression levels. However, patients who had increased expression of phosphor-STAT3 (p-STAT3), phosphor-AKT (p-AKT), or MET had similar PFS to those who did not (Fig. 3A–C). Moreover, patients with decreased E-cadherin expression had similar PFS to patients with preserved E-cadherin expression (Fig. 3D). Finally, the patients without BIM expression had PFS of 25.0 months (interquartile range: 18.7–not reached) than patients with BIM expression (11.9 months, interquartile range: 6.4–22.7) (p < 0.001, Fig. 3E). Immunohistochemical staining of the five biomarkers is presented in Fig. 4.

**Figure 5:**
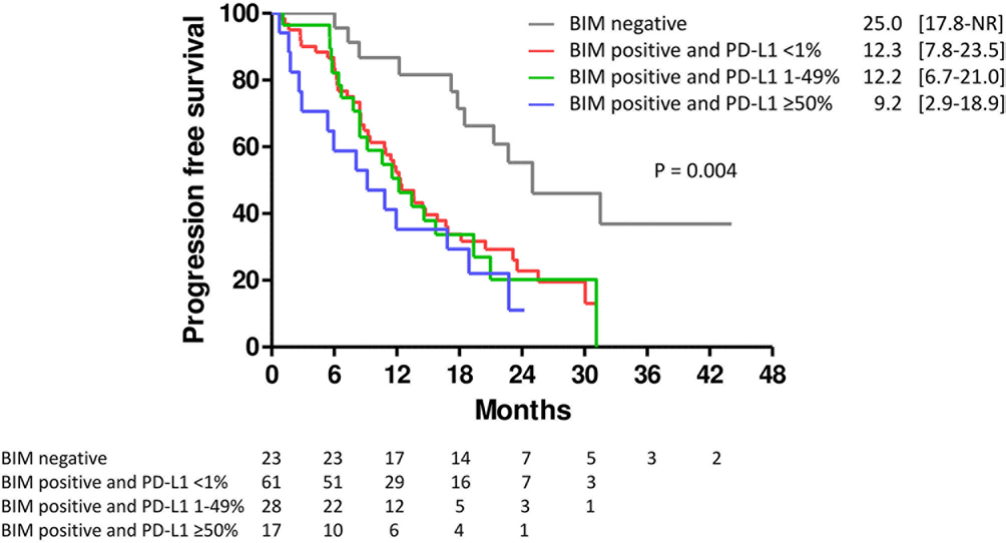
Kaplan–Meier analysis of progression-free survival among patients with different BIM and PD-L1 expression levels. The patients with BIM expression had shorter progression-free survival than those without BIM expression, regardless of PD-L1 expression.

Adjusting the possible confounders by multivariate Cox proportional hazards regression analysis, both the presence of brain metastasis (hazard ratio 1.69, 95% confidence interval: 1.06–2.70; P = 0.028) and BIM expression (hazard ratio 2.94, 95% confidence interval: 1.41–6.14; P = 0.004) were independent poor prognostic factors for PFS (Table 2). Among the 23 patients without BIM expression, 20 were also negative for PD-L1 expression, which was significantly correlated (p = 0.021). Therefore, we further analyzed the PFS of subgroups of patients with BIM expression and found that the PFS was similar among patients with different PD-L1 expression levels (Fig. 5). Moreover, all subgroups of patients with BIM expression had significantly shorter PFS than patients without BIM expression, regardless of PD-L1 expression (p = 0.004) (Fig. 5).

**Table 2:** Cox proportional hazards regression analysis of progression-free survival.

		HR (95% CI)	P-value
Age (years)	> 65 years vs. < 65 years	1.09 (0.63–1.87)	0.759
Sex	Male vs. Female	0.99 (0.63–1.55)	0.963
Brain metastasis	Presence vs. Absence	1.69 (1.06–2.70)	0.028
Performance status	ECOG ≥ 2 vs. 0–1	1.66 (0.85–3.25)	0.138
EGFR mutation	Exon 19 deletion vs. Others	0.97 (0.60–1.57)	0.908
PD-L1 level	≥ 50% vs. < 1%	1.42 (0.73–2.76)	0.300
	1–49% vs. < 1%	1.17 (0.68–2.01)	0.576
E-cadherin	Loss vs. preserved	1.33 (0.69–2.56)	0.393
MET	Presence vs. absence	1.00 (0.62–1.62)	0.989
p-STAT3	Presence vs. absence	0.78 (0.48–1.29)	0.337
p-AKT	Presence vs. absence	1.08 (0.64–1.83)	0.767
BIM	Presence vs. absence	2.94 (1.41–6.14)	0.004

EGFR, epidermal growth factor receptor.

We found that PD-L1 expression was associated with shorter PFS, but with only borderline statistical significance in patients who received first-generation EGFR-TKI (11.5 versus 14.5 months, p = 0.076, Supplementary Fig. 1A). In contrast, BIM expression was associated with shorter PFS (10.9 versus 22.0 months, p = 0.030, Supplementary Fig. 1B) in this patient subgroup. Additionally, both PD-L1 (12.2 versus 25.6 months, p = 0.049) and BIM (12.2 versus NR, p = 0.002) expression was associated with significantly shorter PFS in patients who received second-generation EGFR-TKI (Supplementary Fig. 2A,B). We further adjusted for possible confounding factors using a multivariate Cox proportional hazard regression model. The presence of BIM expression was a poor prognostic factor for PFS, with borderline statistical significance (p = 0.070, Supplementary Table 1) in patients who received first-generation EGFR-TKI; however, it was an independent poor prognostic factor for PFS in patients who received second-generation EGFR-TKI (p = 0.002, Supplementary Table 2). In contrast, high PD-L1 expression was not associated with shorter PFS in patients who received first-generation (p = 0.665) or second-generation (p = 0.887) EGFR-TKI. Although the use of second-generation EGFR-TKI had been associated with a better treatment outcome, BIM expression was still an independent prognostic factor in this patient subgroup in our study (Supplementary Fig. 2B, Supplementary Table 2).

### Assessing apoptotic ratio via flow cytometry

**Figure 6:**
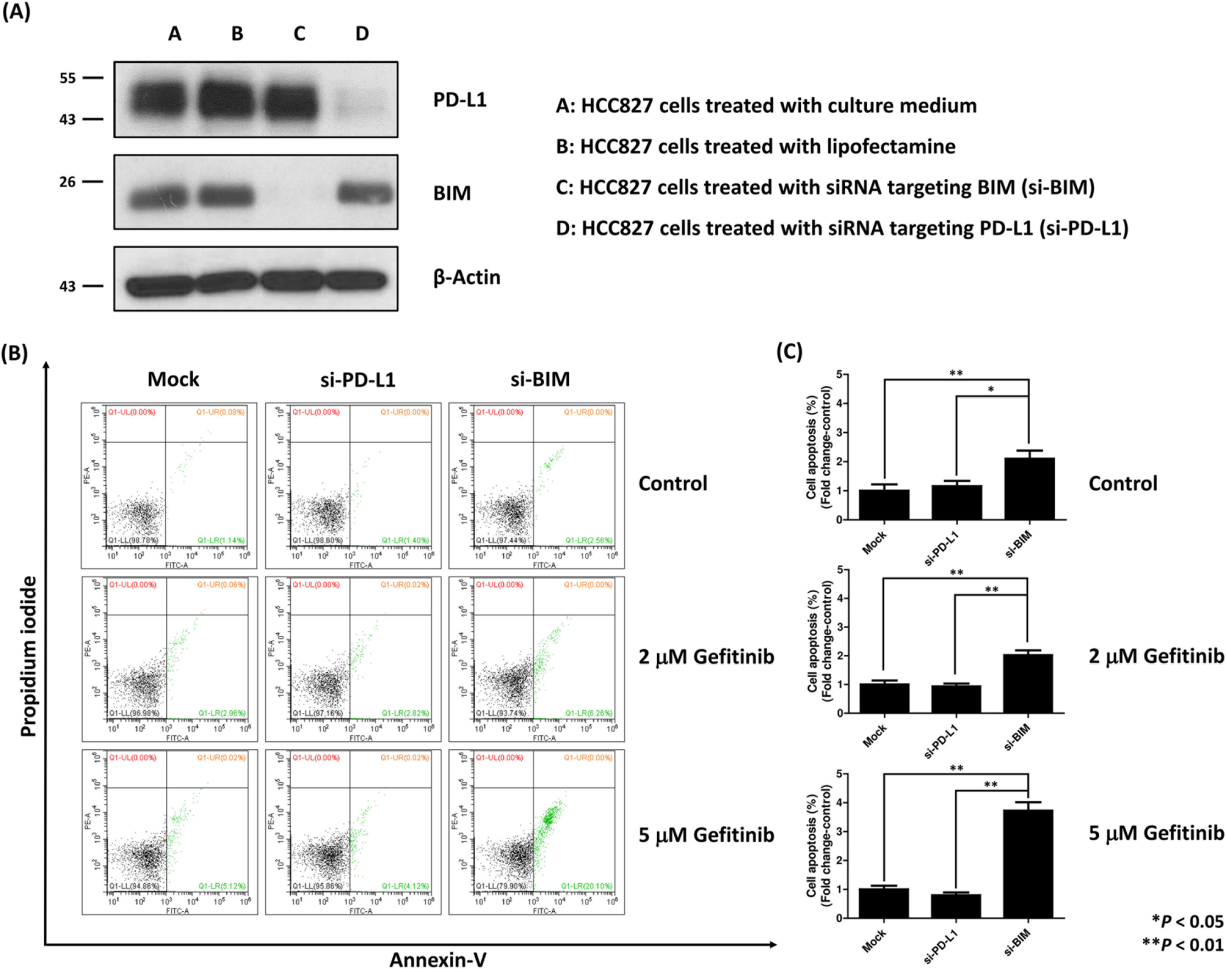
The BIM silencing increased lung cancer cell apoptosis compared to the PD-L1 silencing after treatment with gefitinib. (**A**) The western blot confirmed the silencing of PD-L1 and BIM of HCC827 cells by transfecting siRNA. (**B**) After transfecting with siRNA targeting PD-L1 and BIM, the HCC827 cells were cultured in control conditions (DMSO) or in the presence of the indicated doses of gefitinib, for 24 h. Flow cytometry was performed after staining cells with Annexin V-FITC and propidium iodide (PI). The cells in the Mock group were treated with transfecting vector only. (**C**) Quantitative analysis of Annexin V-FITC flow cytometry showed an increase in apoptotic cells in HCC827 cells with BIM silencing. *P < 0.05, **P < 0.01.

To investigate the role of BIM knockdown on drug sensitivity, flow cytometry of apoptosis was performed after transfecting HCC827 cells with siRNA targeting PD-L1 or BIM. With transfection of siRNA targeting PD-L1 and BIM, the western blotting of PD-L1 and BIM decreased significantly (Fig. 6A). The original blots are presented in Supplementary Fig. 3. After 24 h of siRNA transfection, HCC827 cells were treated with gefitinib for 24 h. The apoptotic rate of HCC827 cells was determined using flow cytometry with annexin V/PI staining (Fig. 6B). The apoptosis rates in cells transfected with siRNA targeting PD-L1 were similar to those in the control group (Fig. 6C), whereas the apoptosis rates of cells treated with siRNA targeting BIM were significantly higher than those in the control group or cells treated with siRNA targeting PD-L1 (Fig. 6C). These results suggested that the knockdown of BIM expression enhanced the drug sensitivity of HCC827 cells to gefitinib.

### Genomic testing of the BIM intronic deletion polymorphism

A previous study demonstrated that the BIM deletion polymorphism reduced the treatment efficacy of EGFR-TKI^40^. To better clarify the role of BIM protein expression on EGFR-TKI treatment efficacy, we collected residual tissue to perform BIM polymorphic genotyping and investigate the association between BIM deletion polymorphism and the protein expression of BIM. There were 42 patients with lung cancer who had sufficient residual tumor tissue for genomic testing. Among them, DNA from six samples did not qualify for subsequent PCR testing. Of the remaining 36 samples, 4 had the BIM deletion polymorphism and 32 had only the wild-type allele (Supplementary Fig. 4). The association between the BIM deletion polymorphism and BIM protein expression is summarized in Supplementary Table 3. Although there was no statistical significance, all the individuals with the polymorphism also had BIM protein expression.

## Discussion

In the present study, we first proved that both BIM and PDL1 expression was correlated with the PFS of EGFR-TKI treatment in EGFR-mutant NSCLC. Using COX proportional hazard regression analysis, we further demonstrated that BIM expression, instead of PD-L1 expression, was an independent poor prognostic factor for PFS (hazard ratio 2.94, 95% confidence interval, 1.41–6.14; p = 0.004). We further verified the proof-of-concept in vitro; the knockdown of BIM instead of PD-L1 using siRNA enhanced gefitinib-induced apoptosis. Our study is the first to globally identify the effectors mediating the predicting role of PD-L1 expression in EGFR-mutant advanced NSCLC patients treated with EGFR-TKIs, which explains the inconsistency of the prognostic value of PD-L1 in previous studies.

Activation of the EGFR pathway has been shown to increase the expression of PD-L1 and other immunosuppressive factors, suggesting a role for EGFR signaling pathway in remodeling the tumor microenvironment. Tumors from patients with EGFR-mutant NSCLC were also found to be PD-L1 positive by IHC analysis^28,41–43^. The association between PD-L1 expression and EGFR-TKI treatment efficacy has been widely studied, but the results have been inconsistent (Table 3)^8–15,19,41–44^. In a retrospective study conducted by Matsumoto et al*.*, high PD-L1 expression deteriorated the treatment efficacy of EGFR-TKIs. This phenomenon was more significant when higher CD8-positive T cell infiltration was observed in the tumor microenvironment^10^, which implied that PD-1/PD-L1 inhibitors might provide clinical benefits in patients with EGFR-mutant NSCLC and high PD-L1 expression. However, the phase 3 open-label CAURAL trial investigated osimertinib plus durvalumab versus osimertinib monotherapy in patients with EGFR-TKI sensitizing and EGFR T790M mutation-positive advanced NSCLC and disease progression after EGFR-TKI therapy^45^. Although the study was terminated early because of high interstitial lung disease in the separate phase Ib TATTON trial, and the objective response rates were 80% in the osimertinib arm and only 64% in the combination arm. The study not only demonstrated no additional benefit of combination immunotherapy in patients with EGFR-mutant NSCLC but also implied the contradictory role of PD-L1 expression in predicting response to EGFR-TKI.

**Table 3:** Summary of data regarding the prognostic role of PD-L1 in treatment efficacy of EGFR-TKIs.

Author	Year	Patient number	PD-L1 antibody	Outcome associated with high PD-L1 expression
D’Incecco et al.^42^	2015	95	Ab58810	Better RR and longer TTP
Lin et al.^43^	2015	56	Ab58810	Better DCR and longer PFS
Tang et al.^41^	2015	64	E1L3N	No association
Soo et al.^11^	2017	90	SP142	Shorter PFS
Yoneshima et al.^14^	2018	71	Dako 22C3	Shorter PFS
Su et al.^12^	2018	101	SP142	Poor RR, shorter PFS, higher primary resistance rate
Hsu et al.^8^	2019	123	SP263	Shorter PFS, higher primary resistance rate
Matsumoto et al.^10^	2019	52	28-8	Shorter PFS
			D7U8C	
Yang et al.^13^	2020	153	Dako 22C3	Poor RR, shorter PFS, higher primary resistance rate
Yoon et al.^15^	2020	131	Dako 22C3	Poor RR and shorter PFS
Kim et al.^44^	2020	69	SP142	No association
			SP263	
			Dako 22C3	
Chang et al.^19^	2021	114	Dako 22C3	No association
Liu et al.^9^	2021	186	SP263	Shorter PFS
Present study	2022	157	Dako 22C3	Shorter PFS
				Associated with BIM expression

DCR, disease control rate; EGFR, epidermal growth factor receptor; PFS, progression-free survival; RR, response rate; TKIs, tyrosine kinase inhibitors; TTP, time to progression.

The recent studies have disclosed cancer cell-intrinsic PD-L1 signals, including the control of tumor growth and survival pathways, stemness, immune effects, DNA damage responses, and gene expression regulation, especially those that are PD-1-independent^20^. Many studies have provided evidence for oncogenic pathway activation and EMT in modulating cancer cell-intrinsic PD-L1 signals. In a cell line study, constitutive activation of the EGFR signaling pathway induced upregulation of PD-L1 expression through the p-ERK1/2/p-c-Jun pathway^46^. The similar phenomenon was observed in the activation of other oncogenic pathways, including ALK^47^, KRAS^48^, and MET^49^. Moreover, the inhibition of downstream AKT and STAT3 signaling can decrease PD-L1 expression in PC9 cells^50^. Similarly, lung cancer samples with EMT phenotypes, defined by decreased E-cadherin expression, also had significantly higher PD-L1 expression than those with epithelial phenotypes^51^. The suppression of EMT by siRNA-mediated ZEB1 knockdown can also suppress PD-L1 expression^52^. Another mechanism associated with cell-intrinsic PD-L1 expression, which may also affect the treatment response to EGFR-TKIs, is the expression of BIM^53^. BIM is a BH3-only protein of the Bcl-2 family that promotes apoptosis by regulating antiapoptotic Bcl-2 proteins^54^ and is an important factor in EGFR-TKI-mediated apoptosis^28^. BIM upregulation is also associated with the expression of PD-L1 in melanoma^55^. However, data regarding the role of BIM expression in the treatment efficacy of EGFR-TKIs remains controversial^27,56–59^. In the post-hoc analysis of the EURTAC trial, patients with high BIM expression had significantly shorter PFS when receiving erlotinib (9.2 vs. 15.0 months, p = 0.02)^56^. This result was further supported by the other two cohort studies^27,57^. In contrast, in an in vitro cell line study, the apoptotic ratio after gefitinib treatment was positively correlated with BIM expression^58^. Moreover, cells treated with shRNA-mediated BIM knockdown were more resistant to gefitinib therapy^59^. To the best of our knowledge, no prior studies globally examined the impact of these pathways (AKT, STAT3, MET, BIM, and EMT), especially their interaction with PD-L1 expression, on the treatment efficiency of EGFR-TKI. Using IHC to detect the activity of these signaling pathways, we found that activation of the oncogenic pathway (AKT, STAT3, and MET) and EMT pathway (loss of E-cadherin) did not affect PFS. (Fig. 3A–D). However, patients with BIM expression had a shorter PFS than those without BIM expression (Fig. 3E), regardless of PD-L1 expression (Fig. 5). COX proportional hazard regression analysis further confirmed that BIM expression is an independent prognostic factor, whereas the expression level of PD-L1 did not affect PFS in multivariate analysis (Table 2). Furthermore, although the use of second-generation EGFR-TKI is associated with a better treatment outcome^60^, BIM expression is still an independent prognostic factor in patients who received second-generation EGFR-TKI (Supplementary Fig. 2B, Supplementary Table 2). To verify the significant role of BIM in mediating the sensitivity to gefitinib, we transfected EGFR-mutant lung cancer cells (HCC827) with siRNA targeting BIM or PD-L1. We found only the knockdown of BIM increased apoptotic cells following gefitinib treatment (Fig. 6), which is compatible with the IHC finding that only BIM expression has an impact on PFS in patients with EGFR-mutant NSCLC receiving EGFR-TKI treatment.

The current study had some limitations. First, this was a retrospective study of patients from a single center. The baseline characteristics of patients with different PD-L1 or BIM expressions were imbalanced. To eliminate potential confounding factors, we performed COX proportional hazard regression analysis to confirm that BIM expression is an independent prognostic factor. The prospective study with balanced characteristics is required to confirm the results of this study. Second, we used IHC staining to evaluate the role of BIM in the treatment efficacy of EGFR-TKIs instead of the BIM deletion polymorphism. The relationship between BIM expression levels and BIM deletion polymorphisms requires further investigation. We discovered that the patients with the BIM deletion polymorphism also expressed BIM, but there was no statistical significance of this correlation due to the limited patient number. However, previous studies regarding the role of BIM deletion polymorphism in EGFR-TKI treatment still had inconclusive results^6,61–63^. Moreover, we have used the HCC827 cell line with an siRNA-mediated knockdown to confirm the role of BIM expression in the treatment efficacy of EGFR-TKI.

In conclusion, we use retrospective cohort study to reveal that patients with high BIM expression had significantly shorter PFS, regardless of PD-L1 expression. Subsequent in vitro studies also demonstrated an increased apoptotic ratio of HCC827 cells to gefitinib therapy after transfection with siRNA targeting BIM. Our data suggests that, among the pathways associated with cell-intrinsic PD-L1 signaling, BIM is potentially the underlying mechanism that affects the prognostic role of PD-L1 and mediates cell apoptosis when receiving gefitinib treatment in EGFR-mutant NSCLC. Further prospective studies are required to validate the results of this study.

## Supplementary Information

Supplementary Information.

## Data Availability

The raw data supporting the conclusions of this article will be made available on reasonable request by the corresponding author, Dr. Po-Lan Su.
