# Supplementary material for: Effect of BIM expression on the prognostic value of PD-L1 in advanced non-small cell lung cancer patients treated with EGFR-TKIs
Source: Sci Rep. 2023 Mar 9;13:3943. doi: 10.1038/s41598-023-30565-4 (PMC9998621; doi:10.1038/s41598-023-30565-4)
Supplement: Supplementary file 1 — Supplementary Information. [file 41598_2023_30565_MOESM1_ESM.pdf]

Effect of BIM expression on the prognostic value of PD-L1 in advanced non-small cell lung cancer patients treated with EGFR-TKIs

Chang-Yao Chu[1#], Chien-Yu Lin[2#], Chien-Chung Lin[2, 3, 4#], Chien-Feng Li[5-7], Shang-Yin Wu[8], Jeng-Shiuan Tsai[2], Szu-Chun Yang[2], Chian-Wei Chen[2], Chia-Yin Lin[9], Chao-Chun Chang[10], Yi-Ting Yen[10], Yau-Lin Tseng[10], Po-Lan Su[2*], and Wu-Chou Su[3, 8, 11]

Supplementary Table 1. Cox proportional hazards regression analysis of progression-free survival among patients who received first-generation EGFR-TKI

		HR (95% CI)	P-value
**Age (years)**	> 65 years vs. < 65 years	1.12 (0.58–2.14)	0.745
**Sex**	Male vs. Female	1.31 (0.72–2.39)	0.375
**Brain metastasis**	Presence vs. Absence	3.12 (1.52–6.38)	0.002
**EGFR mutation**	Exon 19 deletion vs. Others	1.00 (0.50–1.99)	0.996
**PD-L1 level**	≥ 1% vs. < 1%	0.87 (0.46–1.63)	0.665
**E-cadherin**	Loss vs. preserved	1.37 (0.62–3.03)	0.433
**MET**	Presence vs. absence	1.40 (0.76–2.58)	0.282
**p-STAT3**	Presence vs. absence	0.64 (0.31–1.32)	0.226
**p-AKT**	Presence vs. absence	1.15 (0.58–2.27)	0.694
**BIM**	Presence vs. absence	2.28 (0.93–5.58)	0.070

EGFR, epidermal growth factor receptor; TKI, tyrosine kinase inhibitor

Supplementary Table 2. Cox proportional hazards regression analysis of progression-free survival among patients who received second-generation EGFR-TKI

		HR (95% CI)	P-value
**Age (years)**	> 65 years vs. < 65 years	1.35 (0.49–3.70)	0.561
**Sex**	Male vs. Female	0.51 (0.22–1.21)	0.127
**Brain metastasis**	Presence vs. Absence	0.89 (0.36–2.18)	0.798
**EGFR mutation**	Exon 19 deletion vs. Others	0.78 (0.33–1.88)	0.580
**PD-L1 level**	≥ 1% vs. < 1%	1.07 (0.45–2.54)	0.877
**E-cadherin**	Loss vs. preserved	1.20 (0.35–4.08)	0.767
**MET**	Presence vs. absence	0.38 (0.15–0.99)	0.049
**p-STAT3**	Presence vs. absence	0.91 (0.41–2.06)	0.825
**p-AKT**	Presence vs. absence	1.03 (0.41–2.57)	0.949
**BIM**	Presence vs. absence	13.73 (2.62–72.02)	0.002

EGFR, epidermal growth factor receptor; TKI, tyrosine kinase inhibitor

Supplementary Table 3. Association between BIM protein expression and genotyping

	**BIM wild-type (n = 32)**	**BIM deletion polymorphism (n = 4)**
**Positive BIM expression**	27	4
**Negative BIM expression**	5	0

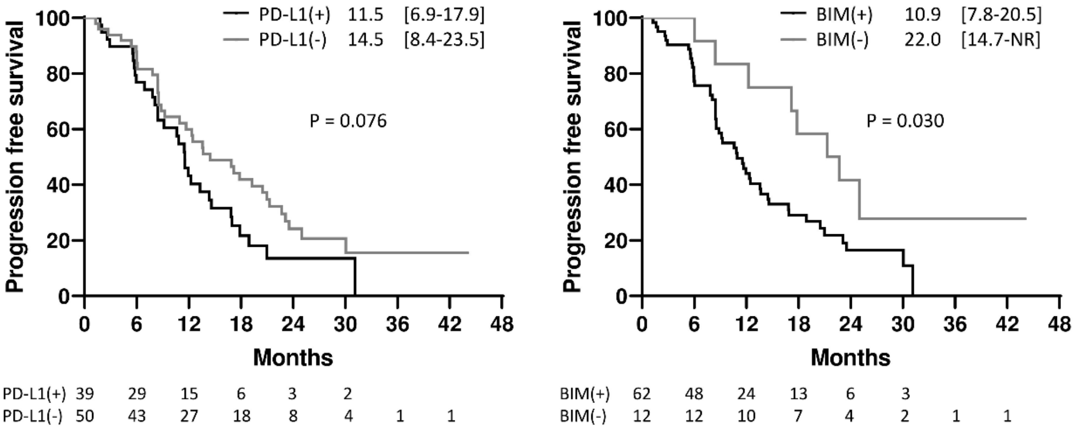

Supplementary Figure 1. Kaplan–Meier analysis of progression-free survival among patients who received first-generation EGFR-TKIs and had different expression levels of PD-L1 (A) and BIM (B).

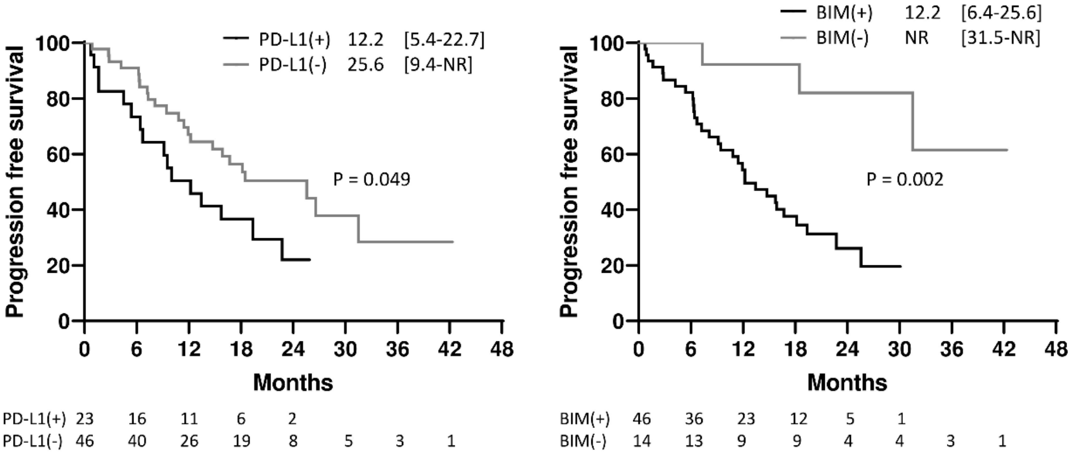

Supplementary Figure 2. Kaplan–Meier analysis of progression-free survival among patients who received second-generation EGFR-TKIs and had different expression levels of PD-L1 (A) and BIM (B).

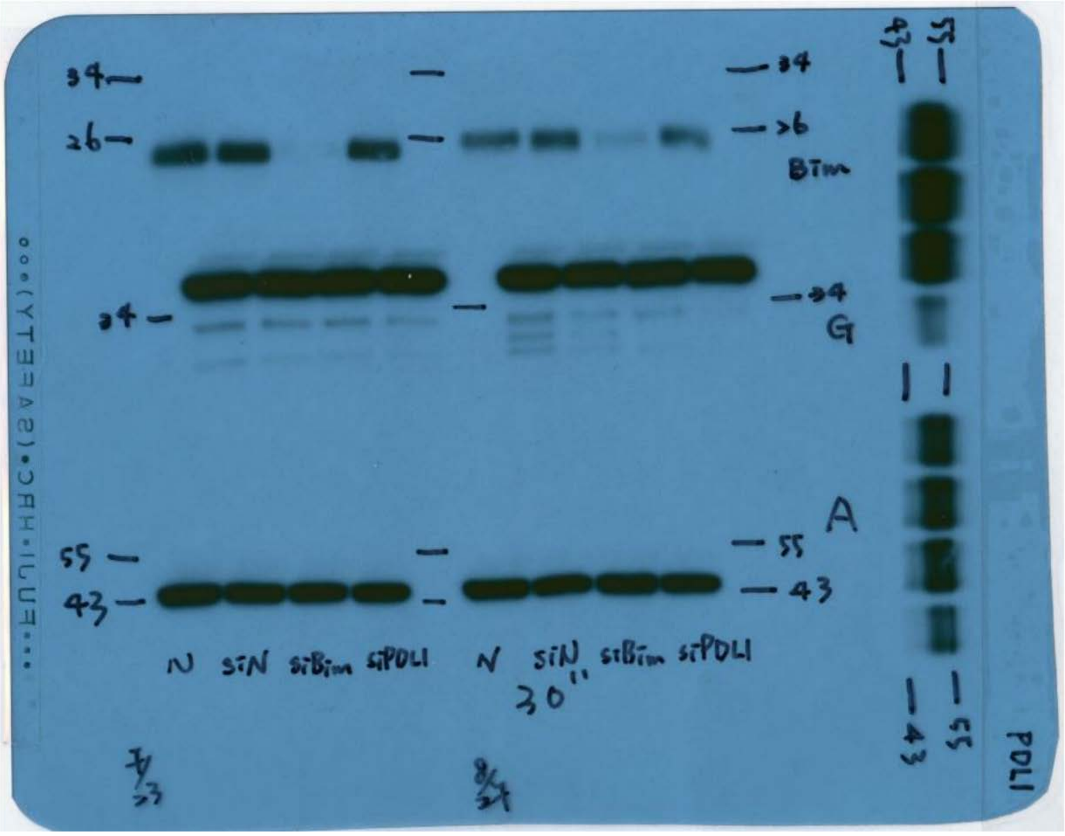

Supplementary Figure 3. The original blots of HCC827 cells with transfection of siRNA targeting PD-L1 and BIM.

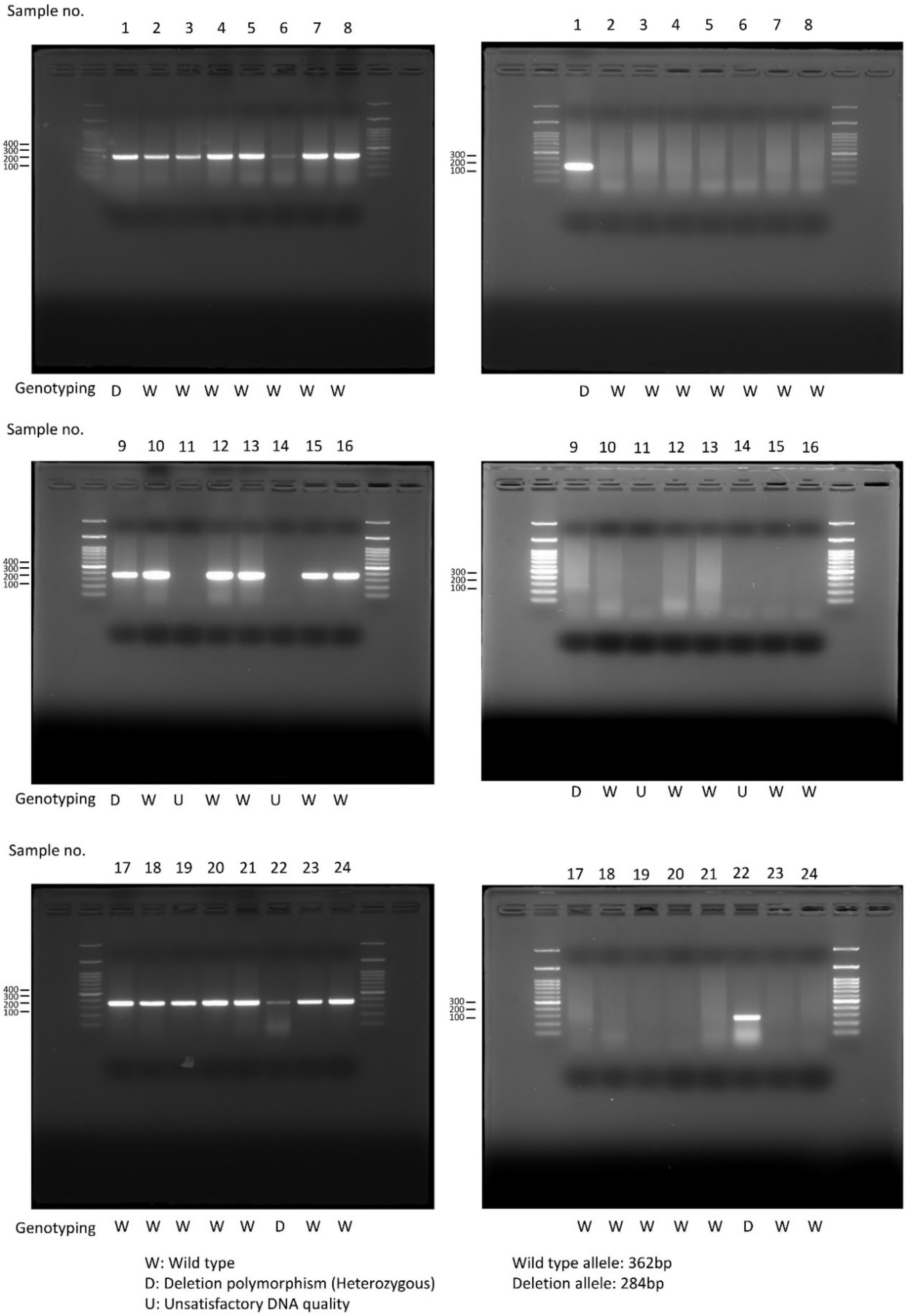

Supplementary Figure 4. Agarose gel images of the PCR products from wild type and BIM deletion alleles. The left side gels analyze the PCR product with wild-type primer, whereas the right side gels analyzed the PCR product with deletion primer.

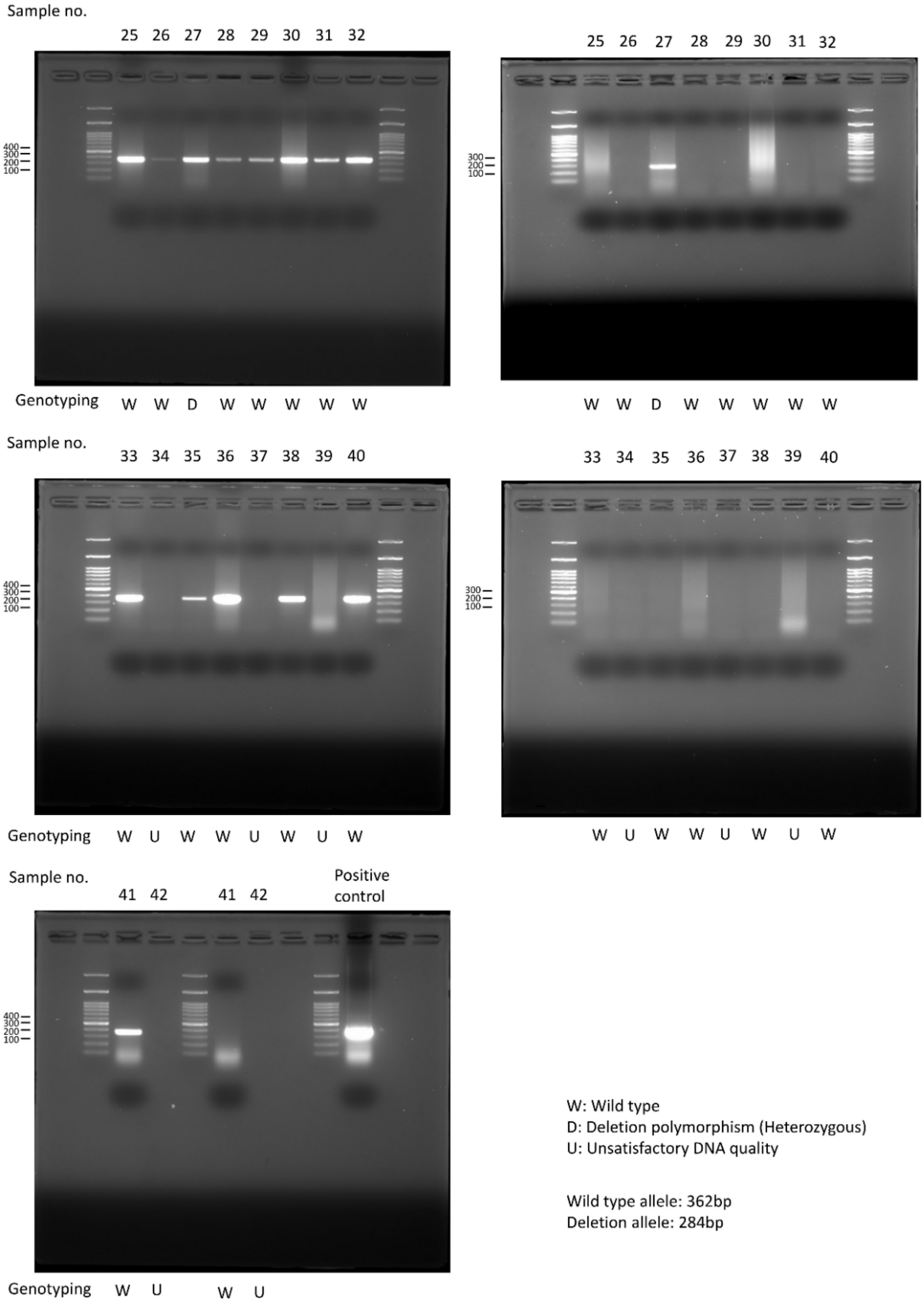

Supplementary Figure 4 (cont.). Agarose gel images of the PCR products from wild type and BIM deletion alleles. The left side gels analyze the PCR product with wild-type primer, whereas the right side gels analyzed the PCR product with deletion primer.
